# Genetic Variations of Glutathione S-Transferase Influence on Blood Cadmium Concentration

**DOI:** 10.1155/2012/356126

**Published:** 2012-01-12

**Authors:** Nitchaphat Khansakorn, Waranya Wongwit, Prapin Tharnpoophasiam, Bunlue Hengprasith, Lerson Suwannathon, Suwannee Chanprasertyothin, Thunyachai Sura, Sming Kaojarern, Piyamit Sritara, Jintana Sirivarasai

**Affiliations:** ^1^Department of Social and Environmental Medicine, Faculty of Tropical Medicine, Mahidol University, Bangkok 10400, Thailand; ^2^Health Office, Electricity Generating Authority of Thailand, Nonthaburi 11130, Thailand; ^3^Excellence Service Center For Medical Technology and Quality Improvement, Faculty of Medical Technology, Mahidol University, Bangkok 10700, Thailand; ^4^Office of Research Academic and Innovation, Faculty of Medicine Ramathibodi Hospital, Mahidol University, Bangkok 10400, Thailand; ^5^Department of Medicine, Faculty of Medicine Ramathibodi Hospital, Mahidol University, Bangkok 10400, Thailand; ^6^Division of Clinical Pharmacology and Toxicology, Department of Medicine, Faculty of Medicine Ramathibodi Hospital, Mahidol University, Bangkok 10400, Thailand

## Abstract

The glutathione S-transferases (GSTs) are involved in biotransformation and detoxification of cadmium (Cd). Genetic polymorphisms in these genes may lead to interindividual variation in Cd susceptibility. The objective of this study was to assess the association of *GSTs (GSTT1, GSTM1*, and *GSTP1* Val105Ile) polymorphisms with blood Cd concentrations in a nonoccupationally exposed population. The 370 blood samples were analyzed for Cd concentration and polymorphisms in GSTs genes. Geometric mean of blood Cd among this population was 0.46 ± 0.02 *μ*g/L (with 95% CI; 0.43–0.49 *μ*g/L). Blood Cd concentrations in subjects carrying *GSTP1* Val/Val genotype were significantly higher than those with Ile/Ile and Ile/Val genotypes. No significant differences in blood Cd concentrations among individual with gene deletions of *GSTT1* and *GSTM1* were observed. *GSTP1/GSTT1* and *GSTP1/GSTM1* combinations showed significantly associated with increase in blood Cd levels. This study indicated that polymorphisms of *GSTP1* combined with *GSTT1* and/or *GSTM1* deletion are likely to influence on individual susceptibility to cadmium toxicity.

## 1. Introduction

Cadmium (Cd) is widely dispersed in the environment and causes various diseases, both in occupationally exposed individuals and in the general population. The general population may be exposed to cadmium through ingestion of food and drinking water, inhalation of particulates from ambient air or tobacco smoke, or ingestion of contaminated soil or dust. The major long-term toxic effects of low-level cadmium exposure are renal injury, obstructive pulmonary disease, osteoporosis, and cardiovascular disease [1]. Cadmium is also a potent human carcinogen and has been associated with cancers of the lung, prostate, pancreas, and kidney [2].

 In human body, Cd is basically taken up by the liver. In the liver, Cd can bind with glutathione (GSH) and be excreted into bile. Moreover, Cd can bind to metallothionein (MT) and be stored. Some Cd bound to MT leaks into the plasma and then is taken up by the kidney. The balance between CdMT and nonbound Cd in renal tissue may be responsible for the degree of Cd nephrotoxicity [3, 4]. Therefore, susceptibility to Cd toxicity among individual may probably be related to genetic variations of Cd-metabolizing enzymes. Glutathione S-transferase (GST) gene is one of the potential candidate susceptibility genes-because it plays a significant role in Cd biotransformation and detoxification [5]. The principal function of GST enzymes is conjugations of hydrophobic and electrophilic compounds with reduced glutathione. The intracellular binding reaction with GSH is catalyzed by the GSTs and leads to stable GSH-metal conjugates being transported out of the cell and excreted via feces and urine [6]. Seven GSTs gene families (*Alpha*, *Mu*, *Pi*, *Theta*, Sigma, *Omega*, and *Zeta*) have been described and genetic polymorphisms have been reported for *GSTM1*, *GSTP1*, and *GSTT1*, resulting in either decreased or altered enzyme activity [7, 8]. *GSTT1* and *GSTM1* are particularly important, because they have a deletion polymorphism resulting in impaired catalytic activity, which is associated with greater sensitivity to toxic compounds. The homozygous deleted (null genotype) of *GSTT1* and *GSTM1* genotypes have been associated with enhanced genotoxicity and are believed to be key factors in determining susceptibility to diseases associated with exposure to xenobiotics such as cancer [9]. Polymorphism of *GSTP1* has been found in the coding region. The variant allele of *GSTP1* Val105Ile encodes valine (Val) instead of isoleucine (Ile) at codon 105 due to a base pair exchange, where G substitutes A. These substitutions were linked to a change in enzyme activity [8]. Polymorphisms in *GSTT1*, *GSTM1*, and *GSTP1* may be associated with increased susceptibility to cadmium toxicity [10].

Given that metabolism and detoxification of cadmium (as an electrophile) is mediated by glutathione and GSTs are involved in the complexation of electrophilic substances with glutathione, it is reasonable to hypothesize that genetic polymorphisms in *GST* genes could result in differences in sensitivity to cadmium.

In recent years, the possibilities of applying molecular techniques to toxicogentics are considerably focused, especially for medical investigations and determinations of factors influencing chemical poisonings. These include biochemical and genetic determinants related to acute and chronic effects. To our knowledge, there are limited data on genetic predisposition influence on cadmium detoxification in the human body. Therefore, this study investigated the association of glutathione S-transferase genes (*GSTT1*, *GSTM1*, and *GSTP1*) polymorphisms and blood cadmium concentration in a nonoccupationally exposed population.

## 2. Materials and Methods

### 2.1. Study Subjects

The Electric Generating Authority of Thailand (EGAT) STUDY was the first cohort study of chronic disease in Thailand, originally designed in 1985 (known as EGAT 1), and mainly covered multidisciplinary researches related to cardiovascular disease (CVD) risk factors such as nutrition and toxicology. The 370 subjects in this study were participants in the third survey of EGAT 2 in 2009 (the first survey started in 1998 and second survey in 2003) [11]. Participants completed a self-administered questionnaire, underwent a physical examination, and provided fasting blood samples. The study was approved by the Committee on Human Rights Related to Researches Involving Human Subjects, Faculty of Medicine, Ramathibodi Hospital, Faculty of Tropical Medicine, Mahidol University, in accordance with the ethical standards laid down in the 1964 Declaration of Helsinki. All participants gave their informed consent prior to their inclusion in the study.

Toxicological profile of heavy metals was investigated in both first and third surveys of EGAT 2, and further genetic analysis started in 2009. Ten milliliters of blood were collected by venipuncture into EDTA and heparinized tubes from each subject and immediately centrifuged at 2000 g. Buffy coat, erythrocytes, and plasma were separated and stored at −20°C until genotyping analyses and biochemical measurement were performed.

### 2.2. Determination of Cadmium in Blood

Whole blood cadmium concentrations were measured by graphite furnace atomic absorption spectrometry (GFAAS) with Zeeman background correction after dilution of the blood (1 : 10) with Triton X-100 solution containing diammonium hydrogen phosphate and nitric acid [12]. The concentration was expressed as micrograms per liter.

### 2.3. Genotype Analyses

The genomic DNA was extracted from the lymphocytes by a modified salting out procedure [13] and frozen at −20°C until analysis. The genetic polymorphisms of *GSTM1*, *GSTT1*, and *GSTP1 *were performed by real-time polymerase chain reaction (real-time PCR) according to the method of TaqMan SNP Genotyping Assays on an ABI 7500 instrument (Applied Biosystems, Foster City, CA, USA), in 96-well format. The TaqMan Assay included the forward target-specific polymerase chain reaction (PCR) primer, the reverse primer, and the TaqMan MGB probes labeled with 2 special dyes: FAM and VIC. The concentrations of probes were 0.04 *μ*M. Amplification of 20 ng of DNA was performed during 40 cycles in a reaction volume of 10 *μ*L. TaqMan Universal PCR Master Mix was used for analysis. Thermocycling conditions were 95°C for 15 seconds follow by 60°C for 1 minute. Information of specific probe and primers are available on the National Cancer Institute's SNP500 database web page at http://variantgps.nci.nih.gov/ [14]. 

### 2.4. Statistical Analysis

Statistical analyses were carried out using the SPSS 16.0 for window software (SPSS, Inc., Chicaco, IL, USA). Cadmium data were log-transformed to achieve normal distribution. The values were expressed as geometric means. The comparisons between variables were examined by the Student's *t*-test and analysis of variance (ANOVA). Genotype distribution was analyzed with *χ* ^2^. The effects of all GSTs gene on blood cadmium were evaluated by linear regression analysis. A *P* value of 0.05 was used as the criterion for statistical significance.

## 3. Results

The subjects of this study consisted of 370 employees of EGAT. The geometric mean of the blood cadmium concentrations was 0.46 ± 0.02 *μ*g/L (with 95% CI; 0.43–0.49 *μ*g/L) (Table 1). The level of blood cadmium in smokers was significantly higher than those in nonsmokers (0.72 versus 0.42 *μ*g/L, *P* < 0.05). There were no significantly difference in blood cadmium concentration between gender and alcohol consumption.

The distribution of genetic polymorphisms and mean of blood cadmium concentration for each genotype are shown in Table 2. All genes were in Hardy-Weinberg equilibrium (*P*-value >0.05). The *GSTT1 *present was the most prevalent genotype found in study population (67.3%). Homozygous variant of *GSTP1 *Val/Val was the lowest genotype in study population (5.1%).

Subjects with GSTP1 Val/Val had significantly higher blood cadmium concentration (0.71 *μ*g/L) than did the *GSTP1* wild-type (Ile/Ile) (0.45 *μ*g/L) and heterozygous variant of *GSTP1* Ile/Val (0.45 *μ*g/L, *P* < 0.05). There were no significant differences on blood cadmium concentration among subjects with *GSTT1* and *GSTM1* deletions.

Three of the putative genes with risk genotypes were examined (Table 3). The linear regression analysis indicated a function of genetic polymorphisms on blood Cd level. In this model, the results of regression coefficient of *GSTT1 Val105Ile* (*β* = 0.59, *P* = 0.034) exhibited strong potential predictors on blood Cd concentration. Considering the significant impact of *GSTP1* variant allele and the very small number of *GSTP1 Val/Val* genotype (*n* = 19), we combined these variant-allele subjects with heterozygous genotype (*n* = 158). Moreover, significant effect of gene combination was observed for *GSTP1* variant allele with *GSTM1* and *GSTT1*. Combined *GSTP1-105 Ile/Val* and *Val/Val* with *GSTM1* and *GSTT1* null genotypes were shown as statistical predictors of blood Cd concentrations (*β* = 0.67, *P* = 0.044 and *β* = 0.72, *P* = 0.038, resp.). However, no other effects of gene combinations were observed on blood Cd levels.

## 4. Discussion

A nonoccupationally exposed population may be exposed to cadmium through ingestion of food and drinking water and inhalation of particulates from ambient air or tobacco smoke. In the present study, we found that the geometric mean of the blood cadmium concentration in a nonoccupationally exposed population was in the acceptable range (<5.0 *μ*g/L) [1]. The result agreed with the previous report in Thai population [15].

Gender, age, and alcohol consumption were no influences on blood cadmium levels among this study population, similar to the study by Batáriová et al. [16]. Cigarette smoking was found to be significantly related to blood cadmium concentration. Smokers had significantly higher blood cadmium concentration than nonsmokers (*P* < 0.05) (Table 1). It could be explained that cigarette smoking was a major source of nonoccupational cadmium exposure because of cadmium in the tobacco. It had been reported that cigarettes contain cadmium in concentrations ranging from 1.56 to 1.96 *μ*g/cigarette [17]. Blood cadmium levels observed in this study group appeared to be similar to those reported in the other studies [15, 16, 18, 19].

Polymorphisms in genes, which involved in metabolism of cadmium, were analyzed including *GSTT1*, *GSTM1*, and* GSTP1 *to explore the genetic susceptibility to cadmium toxicity. In the present study, all tested genotype frequencies were in Hardy-Weinberg equilibrium. The *GSTT1 *present was the most prevalent genotype found in study population (67.3%) (Table 2). Homozygous variant of *GSTP1 *Val/Val was the lowest genotype in study population (5.1%). The null genotypes of *GSTT1* and *GSTM1* were found with a frequency of 32.7%, and 57.6%, respectively, while *GSTP1* Ile/Ile, Ile/Val, Val/Val genotype frequencies were 57.3%, 37.6%, and 5.1%, respectively. The similar genotype frequencies were reported in Thai population [20–22].

The GST gene family is involved in the detoxification of electrophilic compounds by conjugating them with GSH and immediately excreted via the bile or urine. The level of GST expression is a crucial factor determining the sensitivity of cell to toxic chemical [23]. This gene family comprises several genes, and many of them are polymorphic in human. *GSTP1* exhibits a missense mutation at codon 105, which substitutes Val to Ile. The polymorphism in *GSTP1* both amino acid exchanges are in the active site of the enzyme and influence the activity toward different substrates [24]. *GSTM1* and *GSTT1* exhibit deletion alleles, which eliminate enzyme activity [25]. In the present study, subjects with the *GSTP1* homozygous variant (Val/Val) had significantly higher blood cadmium level than did those with Ile/Val and wild-type (Ile/Ile) genotype. The possible explanation would be the substitution of Ile→Val in *GSTP1* change catalytic activity of the corresponding GSTs [25, 26]. The change in catalytic activity may reduce in Cd-GSH conjugates and excretion, which results in cadmium accumulated in blood. Since the variation in *GSTP1* genotype is associated with variation of blood cadmium concentration. Therefore, it could be assumed that *GSTP1* play a more important role in variation of blood cadmium concentrations than the other investigated GST polymorphisms.

With regards to gene-gene interactions, the study showed that* GSTP1 *Val variant allele had significantly associated with higher blood cadmium concentration when taking into account* GSTT1 *null or* GSTM1* null genotypes (Table 3). This could be explained by GST conjugated reactive compounds to GSH prior to their excretion from the body. The deletion of *GSTT1* and *GSTM1* genes reduces the catalytic activity [23], and polymorphism in* GSTP1*, changes catalytic activity of the corresponding GSTs [26]. Therefore, the polymorphisms may affect the conjugation and excretion of cadmium, which results in accumulation of this metal in blood. Moreover, not only a single genetic polymorphism of GSTs genes which may alter the enzyme activity, but also combinations of polymorphism may be more important in determining catalytic activities, expression of enzyme, and interindividual variability of disposition and response to substrates. Therefore, this study indicated that the interaction of *GSTP1* Val variant allele with *GSTT1 *null or *GSTM1* null could affect the elevation of blood cadmium concentration.

## 5. Conclusion

These results suggested that polymorphisms of *GSTP1* and combined genotypes of *GSTP1* with *GSTT1* and *GSTM1* may be responsible for susceptibility to cadmium toxicity. The finding confirms that interindividual variations in blood cadmium concentrations are not entirely attributable to environmental exposure but also genetic background. From the point of view of toxicogenetics, some information of gene-environment and gene-gene interactions is provided by our findings. Further studies are needed to investigate the *GSTT1*, *GSTM1*, and *GSTP1* mutations and their enzyme activities related to cadmium concentrations. Moreover, genetic variations of GSTs and metallothionein in environmentally and occupationally exposed individuals should be considered, particularly in respect of mechanism of Cd-induced toxicity.

## Figures and Tables

**Table 1:** The geometric mean of blood cadmium concentrations in a nonoccupational exposed population.

Characteristics	*n*	Blood cadmium concentration (*μ*g/L)	
		GM ± SE	95% CI
Total	370	0.46 ± 0.02	0.43–0.49
Gender			
male	267	0.46 ± 0.02	0.42–0.50
female	103	0.45 ± 0.03	0.40–0.50
Age (yrs)			
45–55 yrs	144	0.45 ± 0.03	0.40–0.51
>55 yrs	226	0.46 ± 0.02	0.42–0.50
Smoking habit			
nonsmoker	294	0.41 ± 0.02^a	0.38–0.42
Smoker	76	0.72 ± 0.04	0.62–0.85
Alcohol consumption			
Nondrinker	178	0.44 ± 0.02	0.40–0.48
Drinker	192	0.47 ± 0.02	0.43–0.53

^a^ Significantly different from smoker, *P* < 0.05 Student's *t*-test.

GM = Geometric mean.

95% CI = 95% Confidence interval.

**Table 2:** Geometric means of blood cadmium concentrations in a nonoccupational exposed population categorized by different genotype.

Gene	Genotype	Frequency		Blood cadmium (*μ*g/L)
		*n*	%	GM ± SE
*GSTT1*	Null	121	32.7	0.49 ± 0.03
	Present	249	67.3	0.44 ± 0.02
*GSTM1*	Null	213	57.6	0.47 ± 0.02
	Present	157	42.4	0.44 ± 0.02
*GSTP1*-105	Ile/Ile	212	57.3	0.45 ± 0.02
rs1695	Ile/Val	139	37.6	0.45 ± 0.03
	Val/Val	19	5.1	0.71 ± 0.08^a,b^

^a,b^ Significantly different *GSTP1 *from Ile/Ile and Ile/Val genotypes, respectively, *P* < 0.05.

**Table 3:** Regression coefficient for blood cadmium by *GSTP1  Val105Ile* and interaction between *GSTP1  Val105Ile* and GSTT1 and GSTM1.

Genotype	Blood cadmium		
	*n*	*β* (S.E.)^a^	*P* value^b^
*GSTP1 Val105Ile *			
* GSTP1 Ile/Ile *	212	0.27 (0.19)	0.324
* GSTP1 Ile/Val *	139	0.35 (0.22)	0.296
* GSTP1 Val/Val*	19	0.59 (0.39)	0.034
*GSTM1 and GSTP1 Val105Ile *			
* GSTM1 +/GSTP1 Ile/Ile *	96	− 0.14 (0.23)	0.462
* GSTM1 +/GSTP1 Ile/Val and Val/Val *	82	0.32 (0.26)	0.108
* GSTM1 –/GSTP1 Ile/Ile *	126	0.29 (0.17)	0.288
* GSTM1 –/GSTP1 Ile/Val and Val/Val*	68	0.67 (0.46)	0.044
*GSTT1 and GSTP1 Val105Ile *			
* GSTT1 +/GSTP1 Ile/Ile *	121	0.20 (0.13)	0.142
* GSTT1 +/GSTP1 Ile/Val and Val/Val *	89	0.18 (0.21)	0.296
* GSTT1 –/GSTP1 Ile/Ile *	108	0.39 (0.22)	0.103
* GSTT1 –/GSTP1 Ile/Val and Val/Val*	52	0.72 (0.58)	0.038

^a^ Regression coefficients.

^b^ *P* value were obtained by linear regression after controlling for sex, age, BMI, smoking status and alcohol consumption.
